# The Operon as a Conundrum of Gene Dynamics and Biochemical Constraints: What We Have Learned from Histidine Biosynthesis

**DOI:** 10.3390/genes14040949

**Published:** 2023-04-21

**Authors:** Sara Del Duca, Giulia Semenzato, Antonia Esposito, Pietro Liò, Renato Fani

**Affiliations:** 1 Department of Biology, University of Florence, Via Madonna del Piano 6, 50019 Sesto Fiorentino, Italy; 2 Council for Agricultural Research and Economics, Research Centre for Agriculture and Environment (CREA-AA), Via di Lanciola 12/A, Cascine del Riccio, 50125 Firenze, Italy; 3 Department of Computer Science and Technology, University of Cambridge, Cambridge CB3 0FD, UK

**Keywords:** operon evolution, histidine metabolic pathway, piecewise model, thermophily, metabolon

## Abstract

Operons represent one of the leading strategies of gene organization in prokaryotes, having a crucial influence on the regulation of gene expression and on bacterial chromosome organization. However, there is no consensus yet on why, how, and when operons are formed and conserved, and many different theories have been proposed. Histidine biosynthesis is a highly studied metabolic pathway, and many of the models suggested to explain operons origin and evolution can be applied to the histidine pathway, making this route an attractive model for the study of operon evolution. Indeed, the organization of *his* genes in operons can be due to a progressive clustering of biosynthetic genes during evolution, coupled with a horizontal transfer of these gene clusters. The necessity of physical interactions among the His enzymes could also have had a role in favoring gene closeness, of particular importance in extreme environmental conditions. In addition, the presence in this pathway of paralogous genes, heterodimeric enzymes and complex regulatory networks also support other operon evolution hypotheses. It is possible that histidine biosynthesis, and in general all bacterial operons, may result from a mixture of several models, being shaped by different forces and mechanisms during evolution.

## 1. The “Operon Model”: Story of an Idea

In the review article entitled “Genetic Regulatory Mechanisms in the Synthesis of Proteins”, François Jacob and Jacques Monod reinforced contemporary discoveries on genes’ structure and expression patterns into an exhaustive and of great impact theory of gene regulation: the “Operon Model” [1]. This article, published in June 1961 by the Journal of Molecular Biology, can be considered as the starting point in the emergence of a new scientific era [2].

The story of the discovery of the operon concept is a story of passion for science, sharing of ideas, and convergence of (apparently) independent research lines. At one end of a corridor at the Pasteur Institute were André Lwoff, Elie Wollman, and François Jacob. Jacques Monod and his group were at the other end of the hallway. Lwoff worked on lysogenized *Escherichia coli* bacteria able to produce bacteriophage without infection. In the same bacterium, Monod was focusing on the properties of the enzyme β-galactosidase, required for lactose metabolism and synthesized only in the presence of galactosides in the culture medium. As reported by Jacob himself “to all and sundry the two systems appeared mechanistically miles apart. But their juxtaposition would produce a critical breakthrough for our understanding of life, demonstrating that we cannot presume to know how new ideas will arise and where scientific research will lead” [3,4].

In 1957, Jacob, Monod, and the American scientist Arthur Pardee, who was spending a sabbatical year in Monod’s laboratory, performed a crucial experiment that is generally known as PaJaMo, i.e., the initials of the three scientists’ names [5]. The PaJaMo experiment represents the starting point that led to the proposal of a model of negative regulation. Moreover, it generated two other fundamental concepts: the messenger RNA and the operon [5]. In both systems (that of the regulation of the synthesis of β-galactosidase and that of the control of bacteriophage λ lysogeny), they proposed that the product of a regulator gene, the **repressor,** controls and coordinates a group of genes with related functions. This group of genes constitutes an **operon**, and the region on the DNA that responds to the repressor was named **operator**. The repressor can act in *trans*, while the operator functions in *cis* to the operon. In the absence of an inducer, the expression of the genes that constitute the operon is inhibited by the binding of repressor to the operator. Otherwise, when the repressor is induced, it detaches from the operator and the genes are transcribed [2]. Since its conception, this model has been validated various times [5]. The 1961 review article reports and summarizes these experiments and their effects [6]. These papers transformed thinking about gene regulation, introducing for the first time the concept of **regulatory genes**, a new class of genes with no metabolic or structural function, but with the ability to control the expression of metabolic functions [2,7]. The operon model, indeed, described two events: (i) how coding genes’ expression works, and (ii) how this expression is regulated [8].

The ideas presented in these papers were rapidly and widely accepted and welcomed among biologists [2,7], and in 1965, André Lwoff, Jacques Monod, and François Jacob shared the Nobel Prize in Physiology and Medicine “for their discoveries concerning the genetic control of enzyme and virus synthesis” [5,9]. Starting from the beginning of the 1960s, the operon concept matured quickly, and it became manifest that regulatory systems were hugely versatile and plastic. Indeed, it was found out that (i) bacterial genes could be regulated by activators, be subjected to both positive and negative regulations, or be synergistically controlled by combinations of regulatory proteins, that (ii) repressors could also behave as activators, and that (iii) the activity of a given transcription factor often changes depending on the promoter [10].

The idea that the synthesis of bacterial proteins is structured in tangled regulatory circuits was introduced by the operon model. Such circuits could be compared to complex machines control mechanisms, electric circuits, or programs in computers. Indeed, Jacob and Monod can be viewed as promoters of the cybernetics concept in biology [2], as they paved the way for the first synthetic gene networks that, in 2000, introduced the branch of synthetic biology [1].

Many papers have been published in 2011 celebrating the Golden Jubilee year, the 50th anniversary for the publication of Jacob and Monod on the ‘Operon’ concept [2,4,7,10,11,12]. Since 2011, studies on gene organization and regulation have taken place; nonetheless, only a few works on this issue are available in the literature. For this reason, today, 62 years after the discovery of the operon, we believe that the moment has come to take up this concept, in light of the old and the newest scientific discoveries. We will revisit the operon concept from an evolutionary viewpoint; indeed, after Jacob and Monod’s discovery, many models and hypotheses have been proposed to explain the origin and evolution of operon structures. In the present work, we will explore these hypotheses and apply them to a case study, the histidine biosynthetic pathway.

## 2. Definition of Operon

The term operon was first coined by Jacob and Monod in 1961 [6] to describe a cluster of genes whose expression was regulated by an operator. Now, any group of adjacent genes that are transcribed from a promoter into a polycistronic mRNA are defined as operons [13]. All bacterial and archaeal genomes hold operons, and clustered genes with related functions have been reported also for many eukaryotic organisms such as yeasts, fungi, insects, vertebrates, and plants [14,15].

Operons represent one of the principal schemes of gene organization and regulation in prokaryotes [16,17]; about half of all protein-coding genes of a typical prokaryotic genome are organized in multigene operons [18,19], including from two to dozens of genes [20]. They often encode enzymes belonging to the same functional pathway [21], although there are some exceptions such as the Macromolecular Synthesis (MMS) operon, made up of genes involved in replication, transcription, and translation [22]. Moreover, genes in operons often encode proteins that physically or functionally interact, such as enzymes of consecutive steps in metabolic routes ([23] and references therein).

Nevertheless, among prokaryotes, operon conservation is not as common as one would expect [16]. Indeed, prokaryotic genomes are quite unstable [24], and only 5–25% of genes belong to strings shared by at least two distantly related species [25], suggesting that the conservation of operons might be neutral during evolution [24]. Moreover, the operon structure seems to be quite heterogeneous [26], since operons can carry “alien” (genes having homologs in other species but that apparently are not involved in the same metabolic pathway of the other genes of the operon) [26] and/or “ORFan” genes (without homologs in closely related species), and show a different degree of compactness, with closely or widely spaced genes [18].

Most operons are controlled by a single transcriptional promoter situated upstream of the first gene [19]. Nonetheless, many operons are under the control of multiple promoters, regulators, and regulatory sequences [18]. Gene expression can be altered by the organization and order of genes in operons, when specific regulatory mechanisms, such as translational coupling and/or polarity, are involved. Moreover, gene expression increases linearly with the distance from the start of a gene to the end of the operon (“transcription distance”). This is due to (i) a longer time for translation to occur during transcription, and (ii) a six-fold greater translation initiation rate for an mRNA during transcription than after its release, both resulting in an increased gene expression [27].

In the early 1990s, structures similar to canonic prokaryotic operons were found in the genome of the nematode *Caenorhabditis elegans* [28]. Genes in nematode and ascidian genomes are known to be often organized in operons (comprising up to 15–20% of the coding genome) [29] and operons can be horizontally transferred from prokaryotes to eukaryotes [30]. However, the derived polycistronic mRNA is then trans-spliced into monocistronic mRNAs that are individually translated [31].

Recently, numerous computational strategies have been developed to predict operon structures in prokaryotes, based on (i) the intergenic distances between open reading frames (ORFs) of the same operon, (ii) gene cluster conservation among different organisms, (iii) functional relations between genes, since genes in operons often lead to the synthesis of the same protein complex, or enzymes involved in a unique metabolic pathway, (iv) the occurrence of DNA motifs and other sequence elements such as transcription factor binding sites, promoter sequences, and transcriptional terminators, (v) experimental evidences derived from DNA microarray experiments and, more recently, from RNA-seq data, since genes belonging to the same operon are expected to show comparable expression patterns [32,33].

## 3. Hypotheses on the Origin and Evolution of Operons

Operons play a major role in the regulation of gene expression and in the organization of the bacterial chromosome; nonetheless, there is no unanimity yet on why operons are formed and conserved [34]. Three main questions which need to be answered are (i) “Why did operons originate? Which are the possible advantages provided by an operon?”, (ii) “How did operons originate? How did scattered genes cluster during evolution?”, and (iii) “When did operons originate? Are they a recent invention of evolution or were they present in the genome of the last common ancestor (LCA)?”.

Over the years, operon formation has been tentatively explained through various models [23] (Table 1), and they can be split in groups on the basis of the question they want to answer.

### 3.1. Why?

i. The “Fisher model” proposes that gene clusters result from co-adaptation. The physical proximity of co-adapted genes in the genome reduces the probability of recombination events leading to their dissociation and to unfavorable combinations of genes, thus favoring operon assembly [35,43,44].
ii. The “co-regulation model” predicts that genes should be found in operons when their co-regulation would be the most advantageous; indeed, gene clusters promote coordinated expression and regulation ([43], and references therein).
iii. According to the “molarity model”, co-regulation can also ensure that proteins are synthesized in equimolar quantities, thus reducing dissimilarities in their concentration levels [26,45].
iv. In the “selfish operon model”, proposed by Lawrence and Roth in 1996 [37], horizontal gene transfer (HGT) guarantees the spread of functionally related genes organized in gene clusters. The physical closeness of genes does not provide any selective advantages to the single organism but enhances the fitness of the entire gene cluster, as when genes are physically close the probability of a combined transfer of genes increases.
v. Glansdorff [38] proposed that the “adaptation to thermophily” performed a crucial role in the formation of operons. Co-translation of proteins that are functionally related would have facilitated the formation of multienzyme complexes able to channel thermolabile substrates and the mutual stabilization of intrinsically thermolabile proteins.
vi. In accordance with the previous model, the “protein immobility model” (PIM) [39] proposes that gene clustering was pushed by a thermodynamic advantage obtained by the physical closeness of newly translated proteins; in this way the product of one enzyme could easily find its target.
vii. More recently, Nguyen and coworkers [40] developed and used a maximum parsimony algorithm to recreate ancestral operon states. They suggested that two forces, i.e., “the essentiality (the trait of being essential to life) and the formation of a protein complex are two drivers for gene block conservation”. Their idea relies on the analysis of some *Bacillus subtilis* and *E. coli* operons. They also suggested that (i) some operons can quickly and independently evolve in various branches in their taxonomic groups, suggesting that selective pressure plays a key role in the evolution of bacterial operons; (ii) other operons are highly conserved, since their evolution predates the LCA of the investigated clades, (iii) some ancestral operons can be described as intermediate functional structures, and (iv) some operon conservation is occasional, suggesting an involvement of horizontal gene transfer.

### 3.2. How?

i. The “natal model” proposes that operons arose in situ by in-tandem gene divergence and duplication [43], corresponding to the Horowitz “retrograde hypothesis” on the origin and evolution of metabolic routes [41]. According to Horowitz, in the primordial heterotrophic bacteria, various biosynthetic capacities were acquired in a stepwise and sequential enzyme development following a reverse order compared to that of the extant pathways [46]. However, the Horowitz hypothesis has been shown to be relevant in very few cases ([44] and references therein).
ii. Fani and coworkers [13] proposed a “piecewise model” for the origin and evolution of the histidine operon in proteobacteria. According to this model, in the ancestor of proteobacteria *his* genes were initially scattered, coding for monofunctional enzymes; then, they underwent a stepwise compacting process that reached its climax in some γ-proteobacteria.
iii. In the “scribbling pad model”, Norris and Merieau [42] proposed that operon construction could be due to plasmids and integrative conjugative elements. According to this hypothesis, (i) a gene is copied onto a plasmid, (ii) this copy is mutated, (iii) other genes encoding related functions are duplicated and mutated on the plasmid, (iv) these genes are rearranged on the plasmid forming operons, and (v) the resulting operons are transferred back to the chromosome and/or to other bacteria.

### 3.3. When?

A third issue, far less explored than the previous ones, needs to be addressed: “When did operons originate during molecular and cellular evolution?”.

The idea that the organization in operons of genes encoding enzymes involved in the same metabolic pathway was a common rule in prokaryotes that was highly promoted by the discovery that similar operons can be found in microorganisms belonging to different phylogenetic lineages, e.g., *E. coli* and *B. subtilis* [21]. These similarities suggested that the operon organization is an ancient feature that might have predated the LCA [44]. The assembly of genes belonging to the same metabolic pathway might have been evolutionarily advantageous in the early cellular and molecular evolution when, as proposed by Woese [47], there was a high genetic temperature (i.e., instability of the genetic material of the primordial cells) due to the frequent horizontal gene transfers, favoring the interchange of entire metabolic pathways.

The concept of an ancient origin of operons implies that the operon structure should have been in some way dismantled whenever genes involved in the same metabolic pathway are found dispersed along the genome. The comparative analysis of several bacterial, archaeal, and eukaryal completely sequenced genomes evidenced a high variability with substantial rearrangements of gene order among organisms of different phylogenetic lineages [48,49,50,51]. In principle, the extent of gene conservation should be greater within operons than the outer regions, but the comparison of complete microbial genome sequences [24] revealed that their conservation is generally low, highlighting the unstable nature of operons [52]. Therefore, the maintenance of operon structures seems to be of scarce importance, suggesting that their dismantling is almost selectively neutral during long-term evolution. As proposed by Itoh et al. [24], the organization of genes in operon structures can be easily modified during evolution, since the functional constraints against gene co-expression may be very feeble. However, it should be considered that whenever an operon is split in transcriptionally independent units, only the first one retains the regulatory motifs, leading to the possible drastic decrease in the transcription efficiency of the others [24], an event that might affect cell fitness.

The chance that, at least in some cases, the operon organization is evolutionarily recent cannot be a priori precluded. If a specific phylogenetic lineage comprises microorganisms harboring genes of the same metabolic pathway organized in different ways (that is complete gene scattering, compact operons, or partial scattering/clustering) at least two opposite hypothetical scenarios can be delineated to explain this condition:

i. in the genome of the LCA, genes were clustered in operons; this arrangement was then entirely or partly torn down during evolution in some descendants’ branches;
ii. LCA genes were (partially) scattered throughout the genome and the construction of clusters and/or operons occurred in some of the descendants.

The comparative analysis of genes belonging to the same metabolic pathway and arranged differently in organisms belonging to the same or to different phylogenetic lineages might provide some useful clues on the molecular forces/mechanisms that might have guided operon assembly/destruction. This comparison might allow recognition of a formula, if any, in gene organization. From this viewpoint, the histidine biosynthetic pathway constitutes an extremely intriguing case.

## 4. The Histidine Biosynthetic Pathway

L-histidine (His) represents the most active and adaptable natural amino acid, playing roles in protein interactions and often being the central residue in enzyme catalytic reactions [53]. Under physiological conditions, His imidazole side group, with a pKa of approximately 6, allows the amino acid to alternate between the protonated and unprotonated states. Thanks to this feature, His is able to take part in acid-base catalysis; hence, it can be frequently found in the active sites of many enzymes [54]. L-histidine was discovered independently in 1896 by Kossel and Hedin [54], while the study of the His biosynthetic pathway in prokaryotes and lower eukaryotes began in the early 1950s of the last century [55].

L-histidine biosynthesis plays a major role in cellular metabolism and can be defined as a “metabolic cross-road”, being unbranched and interconnected with the de novo synthesis of purines and nitrogen metabolism [56,57]. It is a thoroughly characterized pathway from genetic, biochemical, and evolutionary viewpoints, and its study results are interesting because of (i) the presence of various quite uncommon reactions for a biosynthetic pathway, (ii) the links with other metabolic routes, (iii) the structural characteristics of numerous biosynthetic enzymes, and (iv) the dissimilar *his* gene organization in different organisms ([58], and references therein).

In spite of the different *his* gene organization and structure in different organisms, this biosynthetic pathway is identical in all organisms able to synthesize histidine, including bacteria, archaea, lower eukaryotes, and plants ([59], and references therein). Its deep investigation into *Salmonella enterica* and *E. coli* led to the collection of a consistent body of biochemical, genetic, evolutionary, and physiological data [60]. In these enterobacteria, all the histidine biosynthetic enzymes are encoded by eight adjacent genes (*hisGDC(NB)HAF(IE)*) constituting a single compact operon. The His metabolic route includes bifunctional enzymes (encoded by *hisD*, *hisNB*, and *hisIE*) and a heterodimeric enzyme involved in a single biosynthetic step (encoded by *hisH* and *hisF*) [60], for a total of ten enzymatic steps that convert 5-phosphoribosyl-1-pyrophosphate (PRPP) to L-histidine.

Chemical and biological data suggests that His formed abiotically, being already present on Earth during the long era of abiotic chemical synthesis of organic compounds ([61], and reference therein). Since His plays a key role in metabolism, constituting the catalytic sites of many enzymes [54], if histidine was required in primitive enzymes, the depletion of its prebiotic supply imposed a selective pressure, favoring those organisms able to synthesize this amino acid. The necessity to produce histidine suggests that this biosynthetic pathway is ancient and that it was already part of the metabolic abilities of the LCA [44,61,62]. However, the results of the evolutionary comparison of the *his* genes in the three cellular domains clearly indicate that, after the divergence from the LCA, *his* gene organization, structure, and order have faced extensive reorganizations in the three cell lineages [60,63,64].

## 5. The Histidine Operon as a Model for the Study of Operon Origin and Evolution

Many primary mechanisms in biology were unraveled through the study of the histidine biosynthesis [65], which led to the institution and to the expansion of the concepts regarding the evolution of biosynthetic pathways and modern cell biology [60]. For example, the histidine biosynthetic route was of high importance in the definition and refinement of the operon theory [66,67,68,69], in the study of the phenomenon of polarity [69,70], and in determining the mechanisms at the basis of operon expression [71,72,73,74]. These findings were the cornerstone for the identification and elucidation of the attenuation regulatory mechanism of gene expression, a term first introduced to define regulatory patterns of the histidine operon [75,76], even though attenuation was originally described for the tryptophan operon [77].

The *his* gene structure analyses showed that many different molecular mechanisms were involved in the shaping of this pathway [78], i.e., gene duplication, gene fusion, gene elongation, and horizontal gene transfer, and many of the models proposed for the explanation of operon origin and evolution can be applied to the study of histidine biosynthesis, making this route an exceptional model for comprehending the molecular mechanisms responsible for the shaping of metabolic pathways [56].

### 5.1. The Piecewise Model

The comparative analysis of *his* genes, and the study of their structure and organization, do not seem to support the existence of a fully formed compact *his* operon, similar to the *E. coli* one, very early in evolution. Since the first studies performed on histidine biosynthetic genes, results showed that *his* genes may also be organized in sub-operons (e.g., in *Streptomyces coelicolor* [79], *B. subtilis* and *Azospirillum brasilense* [80,81] or scattered along the chromosomal DNA [82,83]. Comparative analyses of the structure and organization of *his* biosynthetic genes performed on proteobacteria [13], archaea [84], and the Bacteroidota-Rhodothermota-Balneolota-Chlorobiota (BRBC) superphylum [64], highlighted a heterogeneous disposition and organization of *his* genes, i.e., genes assembled in more or less compact operons, sub-operons, or regulons (defined as sets of functionally related genes scattered throughout the genome that can be efficiently co-regulated).

The hypothesis that the *his* operon is ancient and that *his* genes were “operonically” organized in the LCA cannot be a priori ruled out, as predicted by Price et al. [85]; however, the high variability of *his* gene structures and organizations in different organisms strongly suggests that, in the common ancestor of these taxonomic groups and maybe also in the LCA, histidine biosynthetic genes were probably scattered along the chromosome and that the *his* operon is a recent creation of evolution. In accordance with this hypothesis, the analysis of the phylogenetic trees of proteobacteria, archaea, and the BRBC superphylum revealed a gradual clustering of *his* genes during evolutionary time [64].

**Figure 1:**
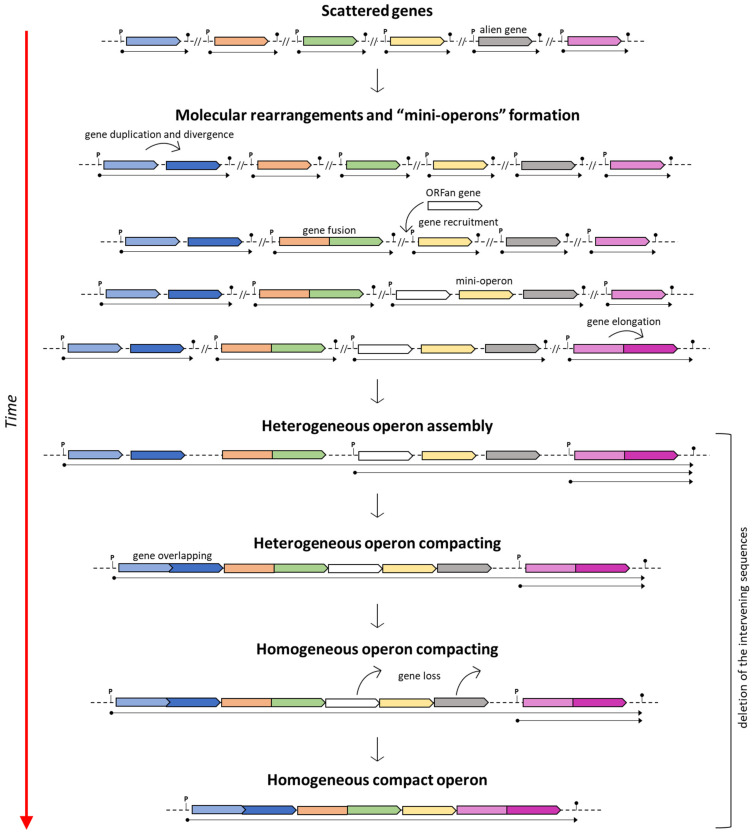
The piecewise model for the operon formation. Adapted from [26].

On the other hand, if the assumption of the ancientness of the *his* operon is true, then various (independent) molecular rearrangements would be necessary to explain this scenario (i.e., the genesis of novel and extremely similar, if not identical, promoter sequences upstream of each separated gene and the separation of genes encoding bifunctional enzymes) [86]. Based on this assumption, Fani et al. [13] suggested that the assembly of compact *his* operons might have arisen through the ongoing clustering of pre-existing sub-operons composed of only some of the genes constituting the ultimate and fully assembled compact operon. This model was proposed to describe the mechanisms involved in the construction of complex operons, and it is known as the “piecewise” model (Figure 1).

### 5.2. The Selfish Operon Model

All the processes that allow the movement of genetic material from one cell to another are referred to as horizontal gene transfer (HGT) [87]. Traditionally, it was believed that limited or no exchange of DNA occurred among diverse life forms and that microorganisms evolved clonally, transferring genes only vertically [88]. That was until the 1950s, when multidrug resistance organisms appeared on a worldwide scale [89].

Today, HGT is a popularly accepted mechanism for adaptation in bacteria and archaea [90] and it is considered a pillar of microbial evolution [87]. Indeed, although duplication events and the resulting paralogous genes are detectable in many bacterial genomes, there is growing proof that bacterial species obtain new genes primarily through lateral transfer [91]. Moreover, it can also be affirmed that not all paralogs in a genome have arisen by gene duplication and divergence within that organism, since homologous genes can also be acquired by HGT [92].

It can be hypothesized that, at the beginning, the early organisms gradually evolved and improved their complexity through HGT, and that lateral transfer was responsible for the distribution of entire metabolic pathways in the bacterial communities, leading to the common ancestors of all the extant organisms [44]. Then, HGT might have been the main driving force behind the evolution and the emergence of the three domains seen today (Archaea, Bacteria and Eukarya) [93].

In nature, the main mechanisms of HGT are transformation, transduction, and conjugation. Other mechanisms contemplate gene transfer agents, membrane vesicles (MV), nanotubes, and cell fusion [87,90,94]. The finding that MV are embedded with DNA fragments representing the entire genome of *S. coelicolor* slightly suggests that also vesicles might be responsible for the introgression of foreign DNA into recipient cells [95].

Although HGT is a continuous process, bacterial genomes are compact and not ever-expanding for the influx of external genetic material, since they continuously undergo the inactivation and loss of genes [91]. Hence, there is an equilibrium between gene acquisition and gene loss. This results in the redefinition of the microorganisms’ ecological niche. Since bacterial genomes are not growing ever larger in dimensions and taking into account the inevitable deletion of genes, it was estimated that HGT has introduced successfully ~16 kb per million years into the *E. coli* genome [89].

**Figure 2:**
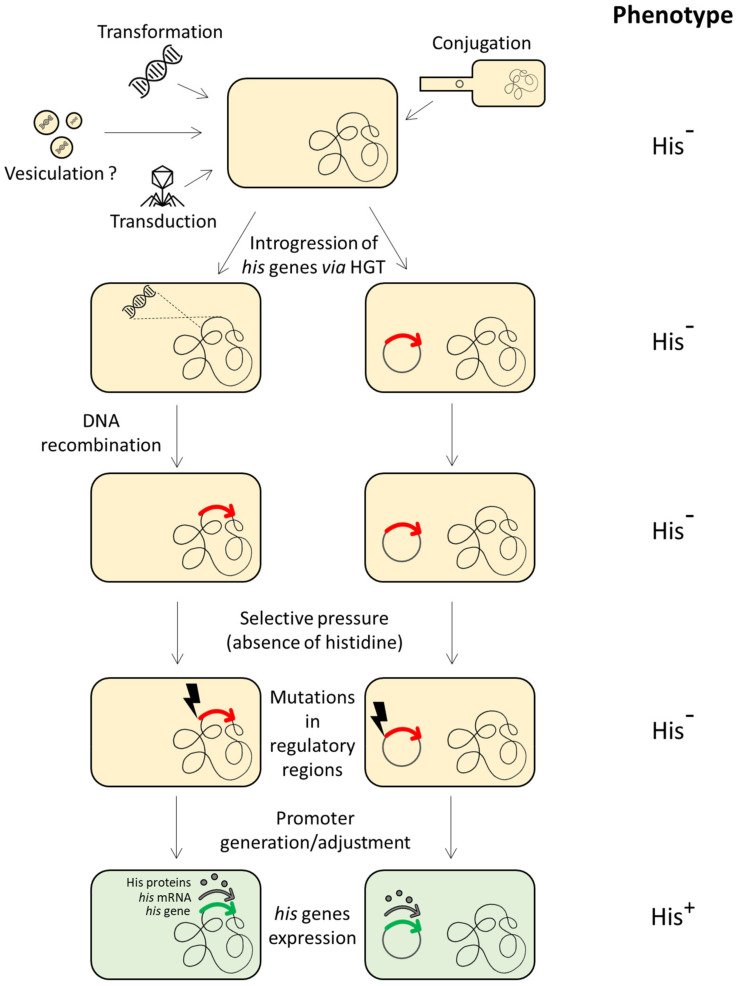
Introgression of histidine biosynthetic genes in a (heterologous) recipient cell through HGT, incorporation into the host genome and fixation via either generation of a new promoter or adjustment of transcriptional regulatory signals. The red arrows represent the external *his* gene when its expression is precluded by the host transcriptional system. The green arrows represent the same gene once expressed by the host cell.

According to the comparative studies performed on proteobacteria, archaea and the BRBC superphylum [13,64,84], evidence suggests the HGT of one or more *his* genes (or the entire operon) among different members of different taxonomic groups. Once the histidine biosynthetic genes introgressed into a heterologous recipient cell (belonging to a different taxonomic group), they can be incorporated into the host genome and fixed by evolution. This requires their expression in the new host, i.e., the regulatory signals should be recognized by the host transcriptional system. However, in principle, the transcriptional signals of the donor *his* genes might not be recognized by the sigma factors of the new host, thus precluding their expression. In spite of this, it has been demonstrated that foreign *his* genes whose transcriptional signals are unrecognized by the RNA polymerase of the host can be expressed under selective pressure by point mutations occurring in a short time scale in the previously unrecognized *his* promoter, thus allowing their expression and fixation by evolution [96] (Figure 2).

According to the selfish operon model proposed by Lawrence and Roth [37], HGT allows the transfer of functionally related genes organized in gene clusters, enhancing the fitness of the cluster itself. These horizontal transfer events, occurred for the *his* operon (or part of it) among members of different taxonomic groups [13,64], might be in agreement with the Lawrence and Roth proposal.

### 5.3. The Interactome Model

For a long time, evolutionary conservation of gene order has been partially attributed to the physical interactions between encoded proteins [52]. Today, it is known that the clustering of bacterial genes into operons reflects an essential co-translational mechanism for a regulation in time and space that is crucial to the successful assembly of protein complexes [97].

The cell is a very crowded environment: macromolecules occupy 20–30% of cellular interiors with a protein concentration of 200–300 mg/mL. The high protein density within the interstitial void results in a gel-like structure, which can alter diffusion processes of enzymes and metabolic intermediates, causing the loss of time and energy necessary for these to come into contact [98,99,100]. As a consequence of molecular crowding and hindered diffusion, it becomes necessary to compartmentalize metabolic routes to surpass diffusive barriers [100]. The structural compartmentalization of the cytoplasm is a well-established concept for the eukaryotic cell [101], while in most prokaryotes the organization of the cytoplasm in bilayer membranes is absent [101]. For a long time and until recently, bacterial cells have been viewed as “bags of enzymes”. Subcellular localization was considered unimportant, and it was thought that bacteria were poorly internally organized and that all the biochemical steps took place in a chaotic way. However, bacterial cells are characterized by a very elevated concentration of macromolecules [102,103,104], resulting in an ineffective metabolism if the enzymes would be translated and localized without any organization. For this reason, most proteins of a living cell are active in complexes rather than in an isolated way [101].

**Figure 3:**
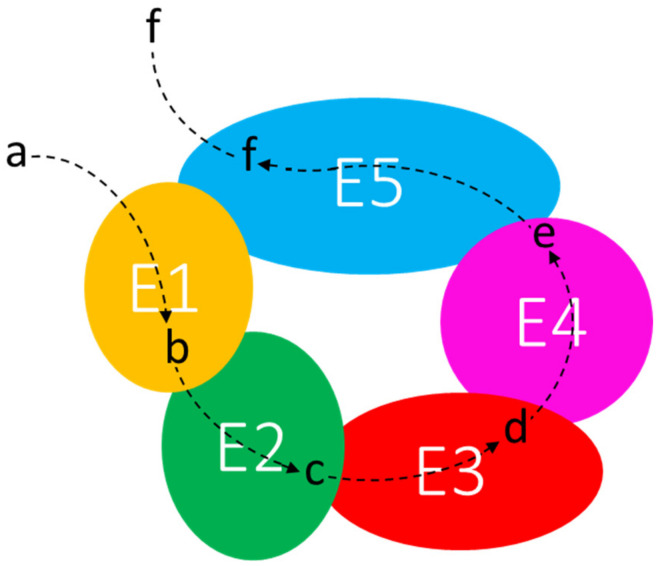
Channeling of metabolic intermediates through the enzymes of a supramolecular complex. E1–E5: enzymes; a–f: substrates and products of enzymatic reactions.

In prokaryotes, the cytoplasm holds many highly ordered structures that represent separate compartments. Multienzyme complexes—whose structural organization may be extremely variable—might constitute the first degree of higher organization of proteins beyond the level of single enzymes [101]. The metabolon is “a complex of sequential enzymes and/or stable multienzyme complexes, which may involve loosely or transiently associated proteins catalyzing sequential reactions of a metabolic pathway” [105]. To refer to these macromolecular transient interactions, the term “quinary structure” was suggested [106]. The specific electrostatic interactions between the enzymes are weak, so that metabolons disrupt during purification techniques. Thus, only a few enzymes were identified as parts of metabolons [101]. One of the main features of the metabolon is its supposed ability to “channel” the intermediates of a metabolic pathway. Channeling implies the favored transfer of a metabolite from one enzyme to a physically close one, with limited diffusion into the surroundings, providing (i) protection of unstable or scarce intermediates, as they are maintained in the protein-bound state, (ii) metabolic advantage by keeping concentration gradients, and (iii) kinetic advantages [99] (Figure 3). Examples of dynamic metabolons have been recently identified in the de novo purine biosynthesis [107] and in the tricarboxylic acid cycle in *B. subtilis* [102]. However, it has been recently reported [108] that “diffusion of metabolites is extremely fast in relation to the rate of catalysis by enzymes, even when the crowded and viscous environment of the cell is taken into account”. The authors conclude that it “is reasonable that diffusion is not the limiting factor for the rate of a reaction and hence metabolite channeling will not increase the rate of the reaction at steady state”. However, in our opinion, translation of proteins in close proximity to each other from a polycistronic mRNA can facilitate the channeling of substrates.

Concerning the organization and structure of the histidine biosynthetic enzymes, it is known that at least seven *his* genes (*hisD*, *hisN*, *hisB*, *hisH*, *hisF*, *hisI* and *hisE*) underwent different fusion events in distinct phylogenetic lineages [78]. Indeed, genes coding for interacting proteins may fuse in prokaryotes [109], thus, fused genes in one organism can indicate a functional, and potentially also physical, association between the independent enzymes in a different organism [110]. Moreover, it has also been suggested that proteins encoded by the *hisBHAF* genes, the highly conserved “core” of histidine biosynthesis, might interact to form a metabolon [61,78,111] (even though this is a hypothesis still not confirmed by experimental evidence), and the physical interaction between the products of *hisH* and *hisF* genes has been recently confirmed by in vivo experiments [112]. This idea agrees with the assumption that genes that encode proteins that need to interact to constitute an active complex are often found to be clustered in conserved operons [23,34].

Thus, these observations connect with the theories about operon formation, especially the PIM [39] and the Nguyen et al. [40] hypothesis. The push towards the organization in operons of the genes of the same metabolic pathway may be, at least in some cases, related to the physicochemical characteristics of the cell cytoplasm and to the thermodynamic advantage obtained through the physical proximity of proteins. Channeling requires that enzymes catalyzing consecutive reactions are colocalized within cells and may temporarily interact to form metabolons. In bacteria, this could be possible when genes are organized in operons: those genes would be transcribed into polycistronic mRNAs, which would be translated into proteins that result in being close to each other [78]. Thus, a specific gene order could be selected because the resulting operon would control the assembly of a multifunctional enzymatic complex [34].

### 5.4. The Adaptation to Extreme Temperatures

The assembly of a supramolecular organization also supports the Glansdorff hypothesis on the origin of operons. In his paper, Glansdorff [38] hypothesized that adaptation to thermophily of the early cells played a major role in gene clustering. Results obtained investigating archaeal lifestyles [84] revealed that, in most cases, thermophilic/hyper-thermophilic Archaea possess *his* genes organized in operons or sub-operons and that most, but not all, mesophilic Archaea possess only scattered *his* genes. Thus, apparently, the adaptation to high temperatures might represent one of the driving forces leading to the organization of *his* genes into operons.

The same was not observed in the case of the BRBC superphylum [64], where the same analysis did not highlight any link between thermophily and the *his* genes organization. Indeed, both thermophilic and mesophilic strains exhibit a huge variety of *his* gene structures. However, the phylogenetic analysis revealed that *his* biosynthetic genes of all the cold-adapted microorganisms are organized in compact and, in some cases, homogeneous operons (i.e., harboring only *his* genes), independently from the organization of *his* genes found in microorganisms of the same phylogenetic lineage. Despite the limited number of cold-adapted species belonging to this group, we propose that the adaptation to low temperature might also have had a role in the organization of His biosynthetic genes, a topic which deserves further analyses.

**Figure 4:**
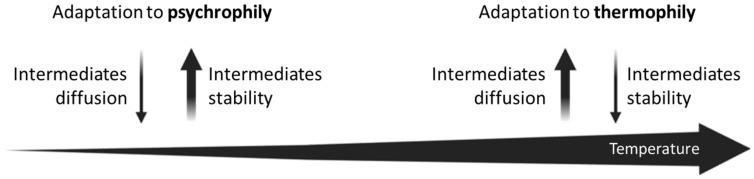
Low and high temperatures as divergent forces leading to the necessity for an operonic gene organization and compartmentalization of enzymes.

Indeed, higher temperatures facilitate the diffusion of metabolic intermediates in the intracellular medium but determine a lower stability of these molecules. Otherwise, lower temperatures allow a better metabolite stability, but at the expense of cytoplasmatic molecular diffusion, due to the limited movements of enzymes and substrates. These two opposite microbial lifestyles could be seen as divergent forces: however, they both lead to the necessity for an operonic gene organization and compartmentalization of enzymes (Figure 4). We are completely aware that the idea of an adaptation to psychrophily is based on the analysis of just one metabolic pathway (i.e., the histidine biosynthesis). Hence, to render this idea a robust statement, a further and deeper investigation of different operons in different taxonomic groups, whose members are characterized by different surviving strategies, is required.

### 5.5. Other Hypotheses

Data reported for the histidine biosynthesis also support some of the other models described above. In detail:

i. The organization and the origin of the gene pair *hisA*-*hisF* supports the natal model [61,113]. Horowitz [114] suggested that the evolution of all genes constituting an operon (and thus, all of the His biosynthetic genes) results from the duplication of a common ancestor gene; however, the analysis of the nucleotide sequence of most of the *E. coli* and *S. enterica his* genes did not reveal any consistent sequence homology between different genes [113]. Despite this, *hisA* and *hisF* originated from a gene duplication event of a common ancestral gene. They both code for (β/α)_8_-barrels and the comparative analysis of the amino acid sequences of HisA and HisF suggested that their respective genes are paralogous and originated from a common ancestor gene through duplication and following evolutionary divergence [113]. For these reasons, HisA and HisF can be viewed as models of retrograde evolution of enzymes in a biosynthetic pathway.
ii. The proximity of *hisH* and *hisF* in the *his* operon/core might be in agreement with the molarity model; indeed, the two enzymes must interact in a 1:1 ratio to obtain the functioning imidazole glycerol phosphate (IGP) synthase, the heterodimeric holoenzyme that links His biosynthesis to both nitrogen metabolism and de novo synthesis of purines [112]. The possibility that also other (if not all) histidine biosynthetic enzymes could physically interact forming a metabolon (see Section 5.3) further supports this evolutionary model.
iii. The existence of multiple sophisticated regulatory systems controlling *his* gene expression supports the co-regulation model. In bacteria and lower eukaryotes, the histidine pathway is controlled by regulatory mechanisms working at the levels of both gene expression and enzyme regulation [65]. One post-translational regulatory system is the histidine-mediated feedback inhibition of HisG [115,116]. Then, at least in *E. coli* and *S. enterica*, histidine biosynthesis can be also regulated at the levels of (i) transcription initiation [60,117,118] thanks to the presence of a primary promoter and two internal promoters, (ii) transcription elongation [60,119,120] through attenuation mechanisms, (iii) transcription termination at the level of cryptic intra-cistronic Rho-dependent terminators [60,121,122,123], and (iv) post-transcription mRNA processing and decay [60,124]. The translational coupling due to the substantial overlap existing between *his* genes and the presence of three genes encoding bifunctional enzymes (*hisD*, *hisNB* and *hisIE*) also reinforce the necessity for coregulation.

## 6. Histidine Genes Order and Biochemical Constraints for Operon Assembly

In those microorganisms in which at least some of the *his* biosynthetic genes are clustered in operons, the relative *his* gene order may differ. However, four of these genes (*hisB*, *hisH*, *hisA*, and *hisF*) are often found in the same relative order (with the exception of archaeal genomes) [84]. These four genes are thought to represent the “core” of histidine biosynthesis, being involved in the central, sequential enzymatic steps of the pathway, and linking histidine biosynthesis with nitrogen metabolism and the de novo synthesis of purines [60,61,111]. Moreover, in proteobacteria and in the BRBC superphylum, the relative order of *his* genes constituting the operons/sub-operons is maintained in all cases [13,64]. According to Tamames [34], the maintenance of gene order can be due to (i) recent divergence of the species, (ii) horizontal gene transfer of a block of genes, or (iii) the importance of the integrity of the cluster to the fitness of the cell. Due to the taxonomic distance between these organisms, and their different ecological niches, it is possible to hypothesize that the importance of the integrity of the cluster could be the principal driver for this specific gene order.

**Figure 5:**
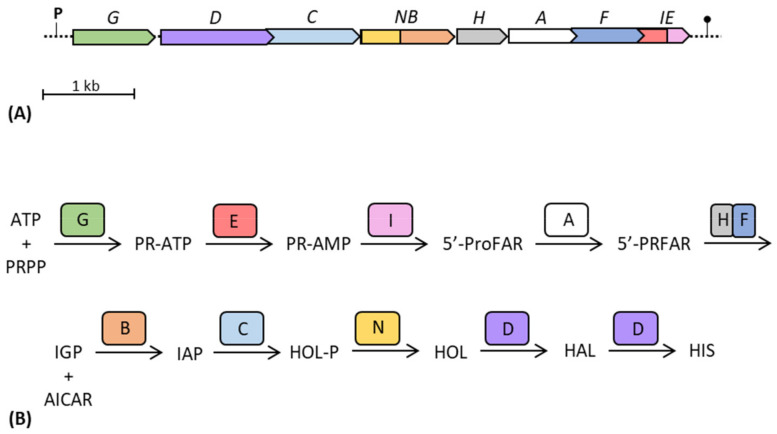
Schematic representation of the gene structure of the *his* operon of *E. coli* (**A**) and of the steps of the histidine biosynthetic pathway (**B**). Adapted from [58]. ATP: adenosine triphosphate; PRPP: 5-phosphoribosyl 1-pyrophosphate; PR-ATP: N′-5′-phosphoribosyl-ATP; PR-AMP: N′-5′-phosphoribosyl-AMP; ProFAR: N′-[(5′-phosphoribosyl)-formimino]-5-aminoimidazole-4 carboxamide-ribonucleotide; PRFAR: N′-[(5′-phosphoribulosyl)-formimino]-5-aminoimidazole-4 carboxamide-ribonucleotide; IGP: imidazole-glycerol-phosphate; AICAR: 5-aminoimidazole-4-carboxamide ribonucleotide; IAP: imidazole-acetol-phosphate; HOL-P: L-histidinol-phosphate; HOL: L-histidinol; HAL: L-histidinal; HIS: L-histidine.

In those organisms in which *his* genes are organized as in enterobacteria, the order of genes in the *his* operon (*hisGDC(NB)HAF(IE)*) apparently does not match the order by which the relative enzymes take part in the synthesis of histidine (HisG, E, I, A, H–F, B, C, N, D) [13] (Figure 5). Indeed, with the exception of *hisG*, which is the first gene of the operon coding the first enzyme of the pathway (involved in the histidine-mediated feedback regulation), all the other *his* genes are located approximately in the opposite order compared to the metabolic reactions catalyzed by their product. It is possible that this specific gene order, followed by specific gene transcription and translation, could allow a certain enzymatic proximity necessary for their physical interaction and the formation of a supramolecular complex. This agrees with the observations of Wells et al. [125]; they showed that operon gene order and organization has been optimized to meet the assembly order of protein subunits, representing an important evolutionary constraint on genes organization. Indeed, the coordination of both timing and location of translation is crucial for maximizing the efficiency of protein complex assembly, and operon gene order has been optimized for the assembly of many protein complexes.

Another possibility, as reported in Section 2, is that the necessity of a different gene expression and regulation imposes a biophysical constraint on the organization of genes in operons and their relative order [27,126].

## 7. When Genes Are Not Organized in Operons

**Figure 6:**
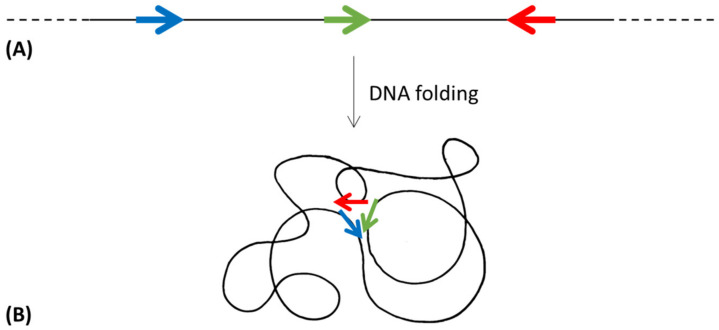
Topological isomerization of a DNA molecule, when allows the spatial proximity (**B**) of the genes involved in the same metabolic pathway and distantly localized in the linear molecule (**A**). Colored arrows represent different genes.

Even though the operon organization of genes can be seen as the winning strategy, genes of the same metabolic pathway often show a high diversity of structures and organizations in many taxonomic groups, with genes organized in more or less compact—heterogeneous or homogeneous—operons, in sub-operons, or in regulons [26]. If, during evolution, some organisms selected a scattered gene organization, there must have been a selective advantage. A possible hypothesis to explain the existence of regulons could be linked to the spatial organization of genes belonging to the same metabolic pathway along the chromosome. DNA is folded to fit inside the cell [127]; however, despite being highly compacted, the nucleoid remains accessible for transcription and replication [128]. Moreover, it must be considered that the bacterial chromosome (even though there is no nuclear envelope separating the nucleoid from the cytoplasm) is not randomly distributed, but it is instead structurally organized [103]. Thus, the DNA folding could allow the physical closeness of the biosynthetic genes and, consequently, the colocalization of the encoded proteins (Figure 6).

The two scenarios in which genes involved in the same metabolic pathway are organized in operons or scattered on the chromosome but spatially close to each other thanks to DNA folding, could be different—but equally (or similarly) effective—strategies to obtain compartmentalization of biosynthetic enzymes in prokaryotes.

In eukaryotes, gene expression is based on individual promoters and monocistronic messages (with few exceptions, i.e., as reported in Section 2, nematode and ascidian genomes [29]). To reach a coordinated expression of functionally related genes, the “RNA operon theory” was proposed [129], stating that mRNAs derived from different chromosomes assemble into ribonucleoprotein particles (RNPs) that act as functional operons (transperons) to give rise to protein clusters. Specifically, transperons are monocistronic mRNAs containing shared *cis* motifs that undergo assembly in *trans* following transcription to form pathway-specific ribonucleoprotein complexes. Chromatin organization seems to be fundamental for their formation, and transperons help facilitate the compartmentalization of proteins into specific complexes created upon cotranslation [130,131].

## 8. Conclusions

Many hypotheses concerning the origin and evolution of operons have been proposed over the years, some of them supported by experimental evidence, i.e., the adaptation to thermophily model, the PIM, the one proposed by Nguyen and coworkers, the scribbling pads, and the piecewise model. Others, such as the natal model, may be invoked to explain only some metabolic routes [44], and the list of known examples of enzymes catalyzing successive steps sharing structural similarities (resulting from a series of gene duplication events) is small ([132], and references therein).

**Figure 7:**
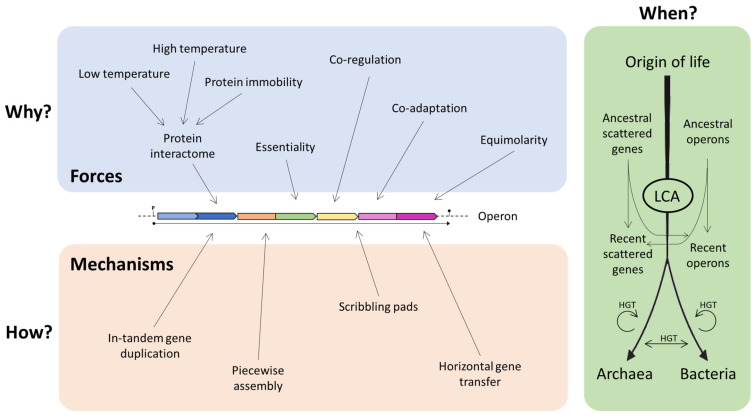
Different forces/mechanisms that contributed to the operon construction. LCA: last common ancestor.

It is possible to imagine that operons may represent the result of the combination of various models, and that even the same operon could have been shaped—during evolution—by different forces and mechanisms [40]. These may depend and vary on the basis of the different environmental conditions in which the organisms live and thrive. It is still not clear which is the contribution of each force/mechanism in the origin of operons, and it is possible that different forces acted separately during evolution (Figure 7).

Concerning histidine biosynthesis, many different models can be applied to its origin and evolution, to explain both how and why histidine complex operons arose, thus reinforcing the idea that different environmental pressures may have led to the organization in operons/sub-operons of *his* genes, and that these complex structures can now be retrieved in taxonomically distant organisms thanks to either HGT or convergent evolution.

## Figures and Tables

**Table 1:** List of the proposed models for the origin and evolution of operons, year of publication, number of citations according to Google Scholar (accessed on 14 February 2023), and “degree of interest” of the model, calculated as the number of citations normalized on the number of years since publication.

Model Name	Reference	Year	N. of Citations	Degree of Interest
Fisher	[35]	1958	26,143	402
Co-regulation	[36]	1960	616	9.8
Molarity	not available			
Selfish operon	[37]	1996	755	28.0
Adaptation to thermophily	[38]	1999	49	2.0
Protein immobility	[39]	2004	17	0.9
Nguyen	[40]	2019	5	1.3
Natal	[41]	1945	800	10.3
Piecewise	[13]	2005	61	3.4
Scribbling pad	[42]	2013	13	1.3

## Data Availability

Publicly available data were used in this study.
